# The Phytohormone Signaling Pathway and Immunity Responses to BYDV Infection in Resistant and Susceptible Oat Cultivars

**DOI:** 10.3390/plants14203229

**Published:** 2025-10-21

**Authors:** Jikuan Chai, Kuiju Niu, Panpan Huang, Wenlong Gong, Yuehua Zhang, Zeliang Ju, Guiqin Zhao

**Affiliations:** 1 College of Pratacultural Science, Gansu Agricultural University, Lanzhou 730070, China; chaijk@gsau.edu.cn (J.C.); niukj@gsau.edu.cn (K.N.); huangpp@st.gsau.edu.cn (P.H.); gongwenlong@caas.cn (W.G.); 2 National Center of Pratacultural Technology Innovation (Under Preparation), Hohhot 010050, China; zhangyh217@yeah.net; 3 Academy of Animal Husbandry and Veterinary Sciences, Qinghai University, Xining 810016, China; juzliang@qhu.edu.cn

**Keywords:** oat, barley yellow dwarf virus, plant–pathogen interaction, MAPK signaling, phytohormone signaling pathway

## Abstract

Oat (*Avena sativa* L.) is a vital cereal and feed crop grown worldwide, but its production is increasingly threatened by barley yellow dwarf virus (BYDV) and aphid infestations in arid and semi-arid regions, particularly in northern China. This study explores the transcriptomic and physiological responses of two oat cultivars MN10253 (resistant) and Qingyin 1 (susceptible) to BYDV at 0, 2, 8, 24, and 48 h post-infection. A combination of phytohormone profiling, differential gene expression analysis, and pathway enrichment was employed to identify mechanisms underpinning disease resistance. Comparative time-course transcriptome analysis revealed 9285 and 8904 differentially expressed genes (DEGs) in MN10253 and Qingyin 1, respectively. Key pathways such as MAPK signaling, plant–pathogen interaction, and hormone signal transduction were significantly enriched. The resistant cultivar exhibited robust activation of pattern-triggered immunity and effector-triggered immunity pathways, marked by upregulation of genes like *RPS2*, *HSP90*, and *WRKY33*, alongside higher expression of salicylic acid (SA)-responsive genes, such as *NPR1* and *PAL.* Conversely, the susceptible cultivar displayed weaker or delayed activation of these defense pathways. Hormonal analysis further demonstrated higher SA accumulation in MN10253 during early infection, correlating with enhanced defense responses. In contrast, Qingyin 1 showed elevated levels of auxin and abscisic acid, which are linked to suppressed immunity. This study underscores the central role of immunity responses and phytohormone pathways in mediating oat resistance to BYDV, highlighting the tradeoff between growth and defense modulated by hormonal crosstalk. These findings advance our understanding of host–pathogen dynamics in oats and provide valuable insights for breeding disease-resistant cultivars.

## 1. Introduction

Oat (*Avena sativa* L.) is a high-quality annual herbaceous crop widely grown in northern China [1]. It is an important feed source in the farming–pastoral ecotone and Tibetan Plateau [2]. The oat growth area provides good roughage for dairy cows and has increased gradually with the development and prosperity of the dairy industry in recent decades. In northern China, the damage caused by barley yellow dwarf virus (BYDV) has been aggravated by global warming, which facilitates the wider spread and increased activity of its aphid vectors [3]. BYDV is one of the most economically important diseases of cereals worldwide. The typical symptoms include plant dwarfing and purplish or reddish coloration of the leaf tips or margins, which extend downward into reddish-greenish stripes or mottling [4]. The presence of aphids on oat leaves worsens these symptoms and leads to yield and quality loss [5]. Low rainfall and a warm, dry climate in the northwest contribute to aphid and BYDV epidemics by favoring aphid overwintering, reproduction, and virulence preservation, which facilitate the spread of BYDV [6]. Therefore, disease control has become crucial in major oat production areas. However, how oats respond to aphid and BYDV infestation remains unclear.

To counter pathogen threats, plants have evolved a highly sophisticated and dynamic immune system that integrates multiple layers of defense [7]. The first line of defense is activated upon recognition of conserved microbial structures, termed pathogen-associated molecular patterns (PAMPs), by cell-surface pattern recognition receptors (PRRs). This response, known as PAMP-triggered immunity (PTI), initiates a broad-spectrum defense involving the production of reactive oxygen species (ROS), mitogen-activated protein kinase (MAPK/MPK) cascade activation, callose deposition, and transcriptional reprogramming of defense-related genes [8,9]. However, successful pathogens can suppress PTI by delivering effector molecules into the host cell, leading to effector-triggered susceptibility [10]. To overcome this, plants have evolved intracellular resistance (R) proteins that recognize specific pathogen effectors, triggering a more robust response known as effector-triggered immunity (ETI) [9]. ETI is often associated with localized cell death at the infection site, termed the hypersensitive response (HR), and the activation of systemic acquired resistance (SAR), which provides long-lasting immunity throughout the plant [11]. Recent evidence indicates that PTI and ETI mutually reinforce each other to fully activate plant immunity, with the MAPK cascade activation playing a pivotal role in this cooperation [12].

In addition, plant immune responses are tightly regulated by a complex network of phytohormones. Salicylic acid (SA) is predominantly associated with defense against biotrophic and hemi-biotrophic pathogens, which require living host tissue for growth. It is a central regulator of SAR, a long-lasting, broad-spectrum immune response that enhances resistance in uninfected tissues [13,14]. SA signaling is also closely linked to localized defense responses, such as HR, which involves programmed cell death at infection sites to limit pathogen spread, characterized by localized cell death and necrosis at the infection sites. In susceptible varieties, typical symptoms caused by BYDV include stunted plants with purple-red or red leaf tips or margins that spread downward into red-green striped or mottled patterns. In contrast, these symptoms are less pronounced in resistant varieties [14]. These processes are mediated by the activation of pathogenesis-related (PR) genes, the accumulation of antimicrobial metabolites, and the production of ROS [15,16]. Other hormones, such as abscisic acid (ABA), brassinosteroids (BRs), gibberellins (GAs), and auxins, also modulate immune responses, either enhancing or suppressing defense depending on the context [17]. ABA, for instance, promotes stomatal closure to restrict pathogen entry; however, it can simultaneously suppress salicylic acid (SA)-dependent defense pathways, thereby modulating plant susceptibility to specific pathogens [18,19]. Similarly, BRs, auxins, and GAs, typically associated with growth regulation, can positively or negatively affect immunity, depending on the context and their interactions with other hormonal pathways [17]. SA intertwines with these phytohormones in plant defense and balance of plant growth and immunity, as well as coordination of responses to biotic stresses [20].

Recent advances in molecular biology and omics technologies have revealed the complexity of hormonal crosstalk and its central role in shaping plant immune responses [21]. Therefore, this study investigates the transcriptomic and physiological responses of two oat cultivars with contrasting resistance to BYDV: MN10253 (resistant) and Qingyin 1 (susceptible). Through an integrative analysis of differentially expressed genes, phytohormone profiles, and enriched signaling pathways, this research seeks to elucidate the molecular mechanisms underlying resistance to BYDV. Special emphasis is placed on key regulatory pathways, including plant hormone signaling, MAPK cascades, and plant–pathogen interaction pathways, which are hypothesized to play crucial roles in mediating immune responses. The findings of this study will enhance our understanding of BYDV–host interactions and contribute to breeding programs aimed at developing resistant oat cultivars with improved disease tolerance.

## 2. Materials and Methods

### 2.1. Materials

‘*Avena sativa cv*. Qingyin 1’ was identified as a BYDV-susceptible variety after years of field trials [22], and ‘*Avena sativa cv*. MN10253’ was determined to be a BYDV-resistant variety [23]. ‘*Avena sativa cv*. Dingyou 4’ was used as an indicator cultivar of the local main strain of BYDV-GPV [24]. Aphids were pathogen-free Schizaphis graminum (Rondani) raised in our lab. Seeds of ‘*Avena sativa cv*. Qingyin 1’, ‘*Avena sativa cv*. MN10253’, and ‘*Avena sativa cv*. Dingyou 4’ were provided by Qinghai University, Gansu Academy of Agricultural Sciences, and Dingxi Academy of Agricultural Sciences, respectively. Aphids with BYDV-GPV specificity were provided by Gansu Academy of Agricultural Sciences. The experiments were conducted in 2021 at the College of Grassland Science laboratory, Gansu Agricultural University.

### 2.2. Aphid Reproduction and Inoculation

Oat seeds of Dingyou 4 were disinfected before being sowed uniformly in plastic boxes (35 × 25 × 15 cm) filled with mixed soil (nutrient soil/vermiculite = 2:1) then placed in an artificial climatic incubator (12,000 lx illumination, 22 °C/18 °C day/night temperature, 60 ± 5% relative humidity, 14 h:10 h (L/D) photoperiod) and watered regularly. When seedlings grew to the three-leaf stage, they were inoculated with pathogen-free Schizaphis graminum nymphs, which grew on the leaves and reproduced for several generations to provide an aphid source.

To reproduce aphids with BYDV-GPV specificity and obtain infected oat leaf samples, another group of plants of cultivar Dingyou 4 were grown using the same method mentioned above. After the plants had grown to the three-leaf stage, they were inoculated by aphids with BYDV-GPV specificity and cultured in the artificial climatic incubator with the same growth conditions mentioned above until the typical symptoms of BYDV developed on the oat leaves.

### 2.3. Oat Plant Preparation and Treatment

Ten seeds of Qingyin 1 and MN10253 were sowed in each pot (10 × 15 cm) filled with mixed soil (nutrient soil/vermiculite = 2:1), with 10 pots for each variety and 3 replicates, making a total of 30 pots per variety. The pots were put in an artificial climatic incubator (12,000 lx illumination, 22 °C/18 °C day/night temperature, 60 ± 5% relative humidity, 14 h:10 h (L/D) photoperiod). After emergence, 5 seedlings with consistent growth were kept per pot. After the plants had grown to the three-leaf stage, pathogen-free aphids were transferred to moisturizing Petri dishes with a brush, which were then sealed with a gauze and put into the incubator at 22 °C to starve the aphids for 12 h. The infected oat leaf samples prepared with the above treatment were then taken and cut into 1–2 cm pieces, put in Petri dishes with filter paper and a moisturizing cotton ball, then inoculated with the starved aphids. The Petri dishes were sealed with gauze and put into the incubator for 48 h at 22 °C in darkness to ensure a complete transmission of BYDV from infected leaves to the aphids.

After the transmission, 10 heads of these BYDV-infected aphids were inoculated on each plant and covered with self-made insect-proof screen. The second and third fully expanded true leaves of 5 plants were collected at 0 h, 2 h, 8 h, 24 h, and 48 h after inoculation (aphids were removed manually with a brush from each plant just before sampling), frozen in liquid nitrogen, and stored at −80 °C for physiological and transcriptomic analyses. Each treatment was replicated three times.

### 2.4. Detection of Phytohormone

Phytohormones in oat leaves were detected by the LC-MS/MS method. Leaves were homogenized with liquid nitrogen. About 0.1 g of tissue powder was suspended in 200 L of pre-cooled water and then used to make a methanol–acetonitrile (2:2, *v*/*v*) extraction. After 60 min of ultrasonic extraction in an ice-bath, the peptide precipitate was incubated at −20 °C and then centrifuged at 14,000 *g* for 20 min. The supernatant was dried by a rotary evaporator, and the residue was resolved in 200 μL of acetonitrile. The precipitate was discarded by centrifuging at 20,000× *g* for 10 min.

The solvent system consisted of water with 0.04% acetic acid (A) and acetonitrile with 0.04% acetic acid (B). The gradient program for pump B started at 5% (0–1 min) then was increased to 95% (1–8 min), maintained at 95% (8–9 min), and finally ramped back to 5% (9.1–12 min). The flow rate was set to 0.35 mL/min, temperature was adjusted to 40 °C, and injection volume was set as 2 μL. Mass spectrometry was conducted in both positive and negative modes using electrospray ionization performed by Analyst 1.6.3 software (AB Sciex). All phytohormones were analyzed in multiple reaction monitoring (MRM) and quantified using Multiquant 3.0.3 software (Sciex).

### 2.5. Transcriptomic Analysis

#### 2.5.1. RNA Extraction, cDNA Library Construction, and RNA-Seq

RNA concentration and purity were measured using a NanoDrop 2000 (Thermo Fisher Scientific, Wilmington, DE, USA). RNA integrity was assessed using the RNA Nano 6000 Assay Kit of the Agilent Bioanalyzer 2100 system (Agilent Technologies, Santa Clara, CA, USA). A total amount of 1 μg RNA per sample was used as input material for the RNA sample preparations. Sequencing libraries were generated using NEBNext UltraTM RNA Library Prep Kit for Illumina (NEB, San Diego, CA, USA) following the manufacturer’s recommendations, and index codes were added to attribute sequences to each sample. Briefly, mRNA was purified from total RNA using poly-T oligo-attached magnetic beads. Fragmentation was carried out using divalent cations at elevated temperature in NEBNext First Strand Synthesis Reaction Buffer (5X). First-strand cDNA was synthesized using random hexamer primer and M-MuLV Reverse Transcriptase. Second strand cDNA synthesis was subsequently performed using DNA Polymerase I and RNase H. Remaining overhangs were converted into blunt ends via exonuclease/polymerase activities. After adenylation of 3′ ends of DNA fragments, NEBNext Adaptors with a hairpin loop structure were ligated to prepare for hybridization. In order to select cDNA fragments of preferentially 240 bp in length, the library fragments were purified with AMPure XP system (Beckman Coulter, Beverly, MA, USA). Then 3 μL USER Enzyme (NEB, San Diego, CA, USA) was used with size-selected, adaptor-ligated cDNA at 37 °C for 15 min followed by 5 min at 95 °C before PCR. Then PCR was performed with Phusion High-Fidelity DNA polymerase, Universal PCR primers and Index (X) Primer. Lastly, PCR products were purified (AMPure XP system), and library quality was assessed on the Agilent Bioanalyzer 2100 system. The raw data of the transcriptome has been uploaded to the NCBI database (PRJNA1186746). The accession link is ‘https://dataview.ncbi.nlm.nih.gov/object/PRJNA1186746?reviewer=8sdqeml9o0ub0oc7l5u8ai02hi (accessed on 8 November 2024)’.

#### 2.5.2. Quality Control

Raw data (raw reads) of fastq format were firstly processed through in-house Perl scripts. In this step, clean data (clean reads) were obtained by removing reads containing adapters, reads containing ploy-N, and low-quality reads from raw data. At the same time, the Q20, Q30, GC-content, and sequence duplication levels of the clean data were calculated. All the downstream analyses were based on clean data with high quality.

#### 2.5.3. De Novo Transcriptome Assembly

To improve the reliability of the sequencing data, the raw image data obtained by sequencing were converted into raw data (or raw reads) by base calling, and then the raw reads were filtered. Containing joints (joint contamination), reads with >5% ambiguous N nucleotides, and low-quality (Q20 > 20%) reads were removed, and then clean reads were obtained. De novo assembly of the clean reads was performed using Trinity with default parameters and then used to cluster transcripts to obtain unigenes [25].

#### 2.5.4. Unigene Functional Annotation and Classification

Unigenes were used as query sequences against the KEGG (Kyoto Encyclopedia of Genes and Genomes); GO (Gene Ontology); NR (Non-Redundant Protein Sequence Database); NT; Swiss-Prot; Pfam; KOG; and TF databases. Annotations of the best hits were recorded.

#### 2.5.5. Identification and Analysis of Differentially Expressed Genes (DEGs)

The expression level of each unigene was calculated and normalized as Fragments Per Kilobase Million (FPKM) values using RSEM software (Version 1.3.3). Identification of DEGs was based on the negative binomial distribution of the DEseq2 package [26]. The cut-off for DEGs was a fold change ≥1 and an adjusted *p* value ≤ 0.001.

### 2.6. Statistical Analysis

The data of the physiological parameters were collected by Microsoft Excel. Statistical analyses were performed using a one-way ANOVA followed by Duncan’s Multiple Range Tests (DMRT) in SPSS version 25.0 and are presented as the means ± standard deviation (SD) from five independent biological experiments.

## 3. Results

### 3.1. Screening of Different Expressed Genes (DEGs)

Transcriptome analysis was completed for 30 samples, yielding a total of 232.98 Gb of clean data. Each sample comprised 5.85 Gb of clean data, with a Q30 base percentage of 92.99% or higher (Table S1). Clean reads from each sample were sequentially aligned against the designated reference genome, with alignment efficiencies ranging from 92.83% to 94.95%.

**Figure 1:**
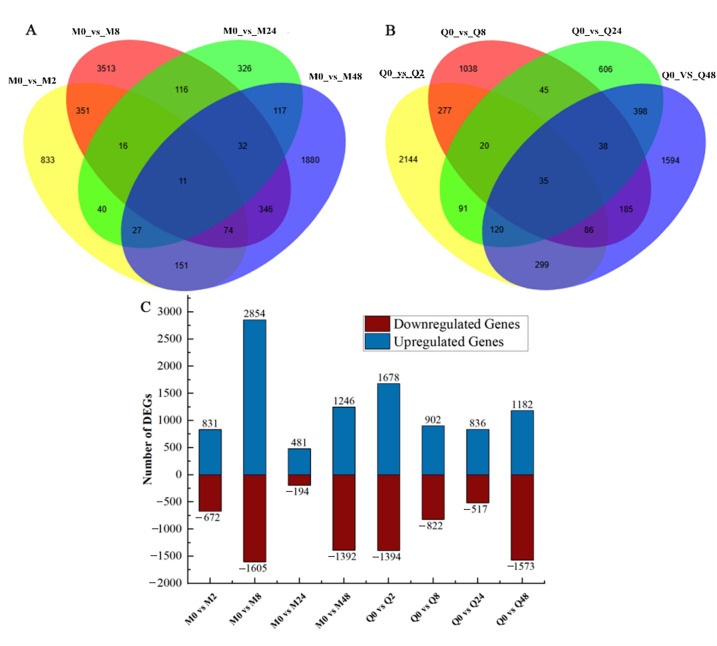
Analysis of differentially expressed genes (DEGs): (**A**,**B**) Venn diagram of differentially expressed genes at different time points in resistant (MN10253) and susceptible (Qingyin 1) cultivars. (**C**) Number of differentially expressed genes between different treatment comparisons in MN10253 and Qingyin 1. Blue indicates upregulated genes, red indicates downregulated genes.

A Venn diagram showed common and uniquely regulated genes in the two cultivars infected by BYDV at four time points (Figure 1). In MN10253, 11 DEGs were identified as common across all treatment comparisons. 833, 3513, 326, and 1880 DEGs were specifically regulated in the M0_vs_M2, M0_vs_M8, M0_vs_M24, and M0_vs_M48 comparison groups, respectively (Figure 1A). In Qingyin 1, 35 DEGs were common across all treatment comparisons, with 2144, 1038, 606, and 1594 unique genes identified in the Q0_vs_Q2, Q0_vs_Q8, Q0_vs_Q24, and Q0_vs_Q48 comparison groups, respectively (Figure 1B). These results highlight interspecific differences between MN10253 and Qingyin 1 in their transcriptional responses to BYDV infestation.

A total of 9285 DEGs were generated after BYDV infection in MN10253, including 3863 downregulated and 5422 upregulated genes. A total of 8904 DEGs were identified in Qingyin 1, with 4306 downregulated and 4598 upregulated genes. In MN10253, the number of upregulated genes was greater than that of downregulated genes in the M0_vs_M8 comparison group. Furthermore, the number of DEGs in this group exceeded the number of DEGs observed across all comparison groups in Qingyin 1 (Figure 1C).

### 3.2. STEM Analysis and GO and KEGG Enrichment of DEGs

**Figure 2:**
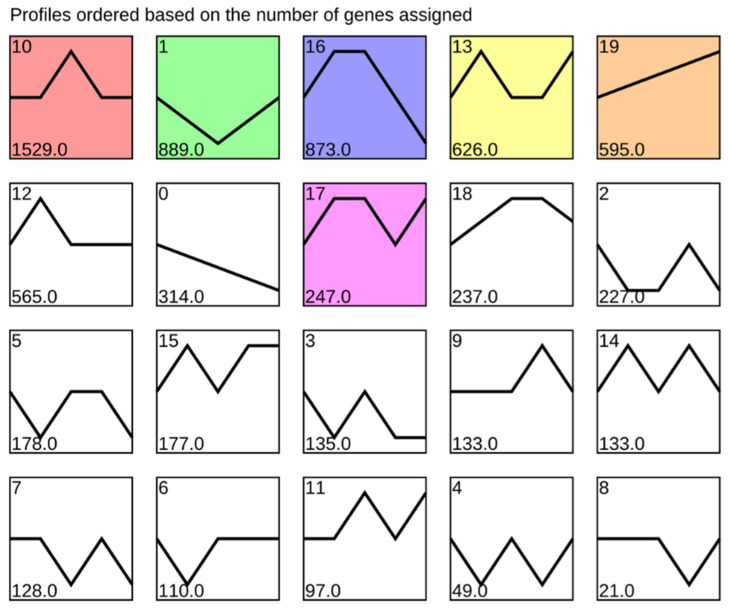
STEM analysis of BYDV response patterns in MN10253. A white background color for a profile indicates no significant change pattern, while other colors indicate a significant change pattern.

According to STEM analysis, genes with the same response pattern to BYDV infection in MN10253 were classified into 20 profiles (Figure 2). Among the seven significant profiles, the most obvious response were profiles 1, 17, and 19. In profile 19, the gene response showed a continuous increase trend after BYDV infection. Genes upregulated at 2 h, 8 h, and 48 h were classed as profile 17, showing a positive response to BYDV infection. However, genes classed as profile 1 showed a negative response within 24 h after BYDV infection.

**Figure 3:**
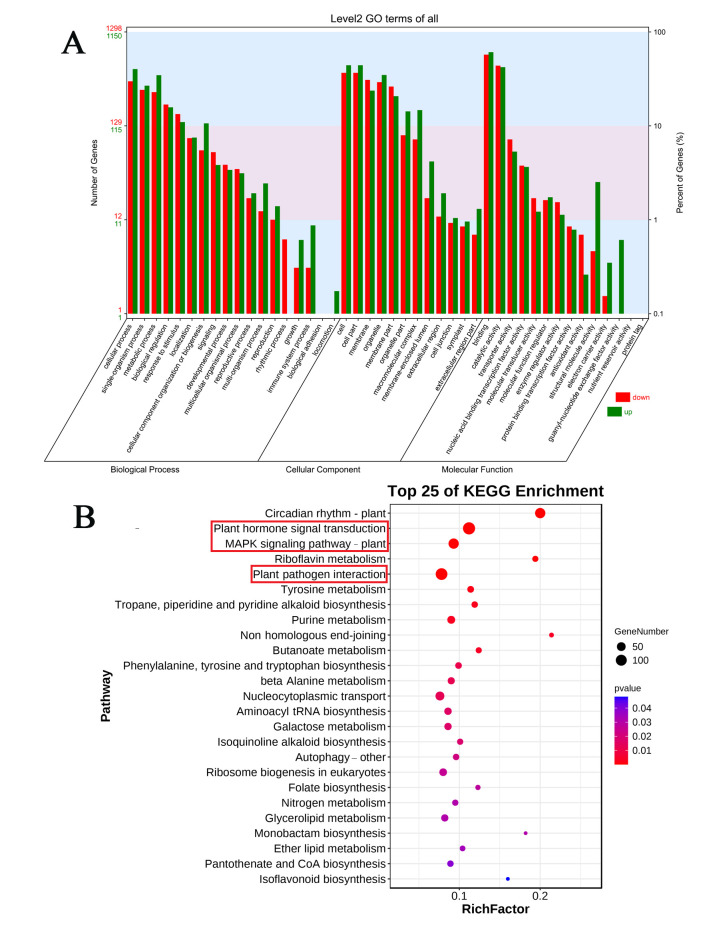
Enriched GO terms and KEGG pathways in the DEGs of profiles 1, 17, and 19 in MN10253 cultivar after BYDV infestation. (A) Bar plots showing the most enriched GO terms in profiles 1, 17, and 19 in MN10253 cultivar after BYDV infestation. (B) Differently DEGs gene in the MN10253 cultivar were analyzed by KEGG enrichment separately in in profiles 1, 17, and 19. The red box indicates the primary enriched KEGG pathways.

To further identify the function of these obvious response genes, GO and KEGG enrichment analyses were performed. According to GO enrichment analysis, DEGs were more enriched in the cellular process, single-organism process, and metabolic process terms of the biological process category. In the cellular component category, DEGs were more enriched in cell, cell part, organelle, organelle part, and macromolecular complex terms. In the molecular function category, DEGs were more enriched in binding, catalytic activity, and structural molecule activity terms (Figure 3B). KEGG enrichment analysis showed that DEGs were mainly enriched in plant hormone signal transduction, the MAPK signaling pathway, and plant–pathogen interaction pathways (Figure 3).

### 3.3. DEGs Related to Plant–Pathogen Interaction

**Figure 4:**
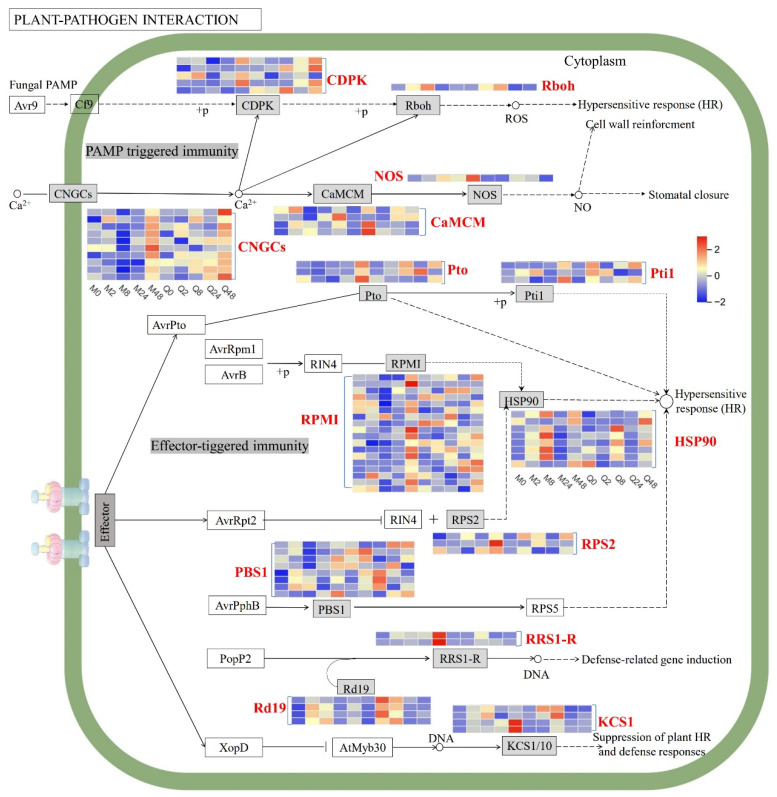
DEGs associated with plant–pathogen interaction pathway. The maximum/minimum log2 FPKM value was set to ±2.0 (red for upregulation, blue for downregulation). Each horizontal row represents a DEG, and the vertical columns represent five points (0, 2, 8, 24, and 48 h) for two genotypes (MN10253 and Qingyin 1).

DEGs involved in the plant–pathogen interaction pathway in both MN10253 and Qingyin 1 were detected, especially the fungal PAMP (pathogen-associated molecular pattern) and effector-related pathway (Figure 4). Several DEGs encoding *CDPK*, *CNGCs*, *Pto*, and *Pti* were significantly upregulated at 48 h after BYDV infection, and several DEGs encoding Rd19 were significantly upregulated at 2 h and 8 h after BYDV infection in both MN10253 and Qingyin 1 cultivars. Several DEGs encoding *RPS2*, *PBS1*, *KCS1*, *RPM1*, and *NOS1* were significantly upregulated at 48 h in MN10253, while they were constant or downregulated in Qingyin 1. More DEGs encoding HSP90 were significantly upregulated at 8 h in MN10253 than in Qingyin 1.

### 3.4. DEGs Involved in MAPK Signaling Pathway

**Figure 5:**
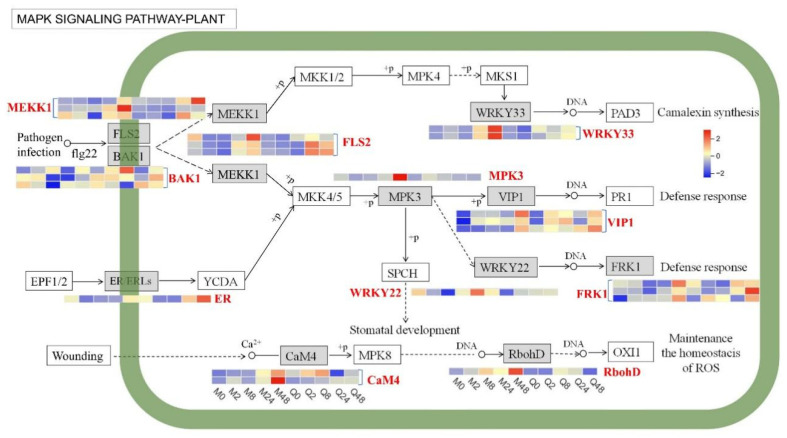
Pathway map showing the differential regulation of the MAPK signaling pathway between MN10253 and Qingyin 1 after BYDV infection. The encoding highlighted in red and blue colors represents up/downregulated.

The KEGG pathway enrichment analysis also indicated that DEGs were enriched in the MAPK signaling pathway (Figure 5). Several DEGs encoding *FLS2*, *MEKK1*, *VIP1*, and *FRK1* were upregulated at 48 h in both MN10253 and Qingyin 1 following BYDV infection. In contrast, several DEGs encoding *WRKY33*, *MPK3*, *WRKY22*, *CaM4*, and *RbohD* were upregulated at 48 h in MN10253, while they were constant or downregulated in Qingyin 1. Additionally, one DEG encoding ER/FRLs was upregulated at 48 h in Qingyin 1 after BYDV infection. Collectively, these findings highlight the differences in gene expression involving in MAPK signaling between MN10253 and Qingyin 1, underscoring the critical role of the MAPK signaling pathway in determining resistance or susceptibility to BYDV.

### 3.5. DEGs Involved in Phytohormone Signal Transduction

**Figure 6:**
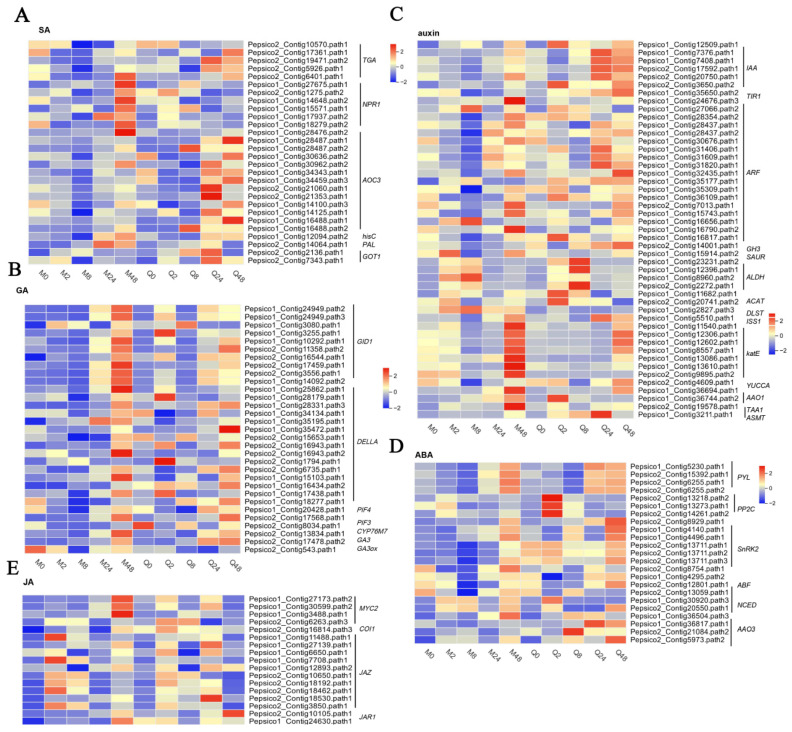
DEGs involved in phytohormone signal transduction pathways in (**A**) salicylic acid (SA), (**B**) gibberellin (GA), (**C**) auxin (AUX), (**D**) abscisic acid (ABA), and (**E**) jasmonic acid (JA). Heat map representing the expression values of DEGs associated with hormone metabolism at eight groups (red for upregulation and blue for downregulation).

In the phytohormone signal transduction pathway, a total of 129 DEGs associated with significant enrichment of SA, auxin, GA, and ABA in oat leaves in response to BYDV infection were identified and analyzed (Figure 6). In the SA signal pathway, only one DEG encoding *TGA* or *AOC3* was significantly upregulated at 48 h in MN10253, while several DEGs encoding *TGA* or *AOC3* were upregulated at 24 h and 48 h in Qingyin 1. Several DEGs encoding *NPR1* were significantly upregulated at 48 h in MN10253, while their expression remained constant in Qingyin 1. Additionally, one DEG encoding *PAL* was significantly upregulated at both 24 h and 48 h in MN10253, but not in Qingyin 1.

For the GA signaling pathway, several DEGs encoding *GID1* and *DELLA* were remarkably upregulated at 48 h in MN10253, with levels notably higher than those in Qingyin 1. In contrast, only one DEG encoding *GID1* was upregulated at 2 h in Qingyin 1 following BYDV infection, suggesting that the response to BYDV in the disease-resistant line MN10253 is more prominent during the later stages of infection (Figure 6B).

In the auxin signaling pathway, several DEGs encoding *AFR*, *katE*, and *TAA1* were remarkably upregulated at 48 h in MN10253, with levels higher than those observed in Qingyin 1. Conversely, several DEGs encoding *IAA* and *ASMT* were significantly upregulated at 24 h in Qingyin 1, with levels higher than in MN10253. Several DEGs encoding *TIR1*, *ARF*, and *YUCCA* were remarkably downregulated at 8 h in MN10253, with levels lower than those observed in Qingyin 1 (Figure 6C).

For the ABA signaling pathway, several DEGs encoding *PP2C* and *AAO3* were upregulated at 2 h in Qingyin 1, with higher expression levels than in MN10253. Furthermore, several DEGs encoding *AAO3* were markedly upregulated at 8, 24, and 48 h in Qingyin 1, while their expression remained constant or was downregulated in MN10253 (Figure 6D).

In the JA signaling pathway, several DEGs encoding *MYC2* and *JAZ* were remarkably upregulated at 48 h and 2 h in MN10253, with levels higher than those observed in Qingyin 1. Conversely, several DEGs encoding *JAR1* were significantly upregulated at 24 h and 48 h in Qingyin 1, with levels higher than in MN10253 (Figure 6E).

### 3.6. Effect of BYDV Infection on Phytohormone Content

**Figure 7:**
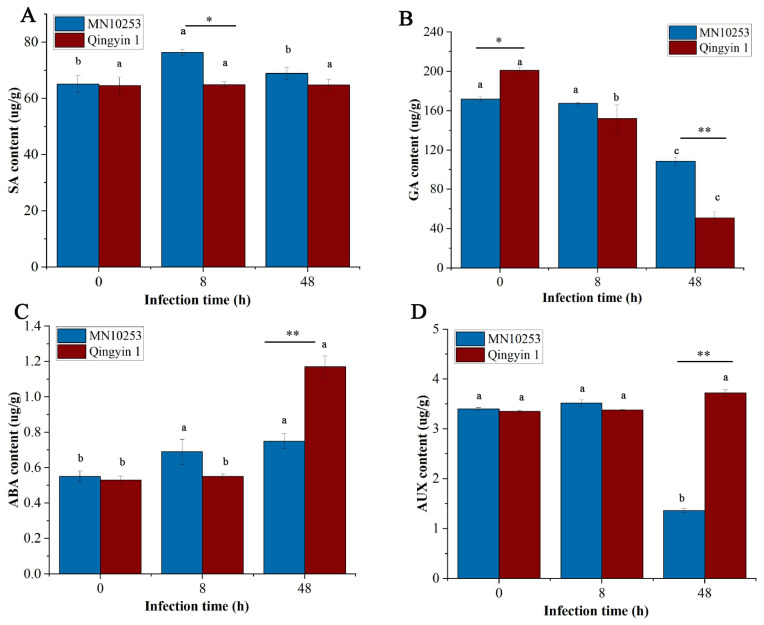
The content changes of plant hormones in (**A**) salicylic acid (SA), (**B**) gibberellin (GA3), (**C**) abscisic acid (ABA), and (**D**) indole-3-acetic acid (IAA) at different stages of BYDV infection. Data are mean ± SE of three independent experiments. Different lowercase letters indicate significant differences between different times for the same material (*p* < 0.05). Asterisks (**) indicate a highly significant difference between the two varieties at the same time point, *p* < 0.01; an asterisk (*) indicates a significant difference between the two varieties at each treatment time point, *p* < 0.05.

Compared with the control, the SA content in MN10253 showed an initial increase followed by a decrease after BYDV infection, with SA levels in MN10253 being higher than those in Qingyin 1 at 8 h. In contrast, the SA content in Qingyin 1 showed no significant changes, indicating that SA might play a sensitive role in the susceptible variety Qingyin 1 (Figure 7A).

As shown in Figure 6B, the GA3 content displayed a decreasing trend in both MN10253 and Qingyin 1 following BYDV infection. At 0 and 48 h, the GA3 content in MN10253 was higher than that in Qingyin 1, suggesting that differences in GA3 content between the two genotypes at these time points could be associated with their intrinsic characteristics.

No significant differences in IAA or ABA content were observed between the two genotypes at 8 h. However, at 48 h, the IAA and ABA contents in Qingyin 1 were significantly higher than in MN10253 (Figure 7C,D). These time points may represent critical stages in the resistance differences between the two genotypes.

## 4. Discussion

### 4.1. BYDV Triggers Immunity Response in Oat

The plant–pathogen interaction pathway is a critical component of plant immunity, comprising pattern-triggered immunity (PTI) and effector-triggered immunity (ETI) [12]. In this study, the differential expression of key genes involved in this pathway highlights their roles in conferring resistance to BYDV in two oat cultivars. Notably, several DEGs encoding calcium-dependent protein kinases (*CDPKs*) and cyclic nucleotide-gated ion channels (*CNGCs*) were significantly upregulated at 48 h post-infection in both cultivars. CDPKs mediate calcium signaling, translating pathogen-induced calcium fluxes into downstream immune responses [27], while CNGCs facilitate calcium influx, critical for ROS production and defense signaling [28]. The upregulation of these genes suggests that both cultivars engage in PTI upon pathogen recognition. However, a NOS1 gene was significantly upregulated at 48 h in MN10253 but downregulated in Qingyin 1. NOS1 encodes a nitric oxide synthase that facilitates the production of nitric oxide—a signaling molecule essential for defense responses such as ROS generation and the hypersensitive response [29]. The higher expression of *NOS1* in MN10253 likely contributes to its superior resistance.

ETI-associated genes further differentiate the two cultivars. DEGs encoding Rd19, a cysteine protease involved in programmed cell death and pathogen defense [30], were upregulated early (2 h and 8 h) in both cultivars. Its early upregulation suggests a role in facilitating localized cell death to restrict pathogen spread [31]. However, key ETI-related genes such as *RPS2*, *PBS1*, *KCS1*, and *RPM1* showed significant upregulation only in MN10253 at 48 h post-infection, while their expression remained unchanged or decreased in Qingyin 1. These genes encode nucleotide-binding leucine-rich repeat (NLR) proteins and other critical ETI components [32,33], thereby activating ETI and robust immune responses. Additionally, HSP90, a molecular chaperone that stabilizes and activates resistance (R) proteins involved in pathogen recognition [34], exhibited stronger upregulation in MN10253, emphasizing its robust ETI response. Collectively, the distinct expression profiles of PTI- and ETI-related genes underscore the divergent defense strategies employed by the two cultivars. The robust immune activation in MN10253 highlights its effective resistance to BYDV infection, whereas the comparatively weaker induction of these defense pathways in Qingyin 1 correlates with its observed susceptibility.

### 4.2. MAPK Signaling Pathway Activation in Response to BYDV in Oat

The MAPK signaling pathway, a conserved cascade in plants, plays a central role in stress responses, including those to pathogens [35]. In this study, upstream components of the MAPK signaling pathway such as *FLS2* and *MEKK1* were upregulated at 48 h post-infection in both cultivars, indicating a conserved initial PTI activation. *FLS2* is a well-characterized receptor-like kinase that recognizes bacterial flagellin and activates downstream signaling cascades, including MAPKs [36]. Similarly, *MEKK1* is a key component of the MAPK cascade that amplifies defense responses upon pathogen recognition [37]. The upregulation of these genes in both cultivars suggests that the initial activation of PTI is largely conserved, regardless of the resistance phenotype.

In contrast, downstream components of the MAPK signaling pathway, including *WRKY33*, *MPK3*, *WRKY22*, and *RbohD*, were significantly upregulated at 48 h post-inoculation in the resistant cultivar MN10253, whereas their expression either remained constant or was downregulated the susceptible cultivar Qingyin 1. *WRKY33*, a transcription factor critical for plant immunity, regulates the activation of defense-related genes [38]. *MPK3*, encoding a key MAP kinase, is known to activate WRKY33 and other downstream components, facilitating the accumulation of defense hormones such as salicylic acid [39]. Furthermore, *RbohD* plays an essential role in ROS production by encoding an NADPH oxidase, which acts as a pivotal signaling molecule in plant defense responses [40]. The robust expression of these genes in MN10253 highlights the enhanced capacity to mount an effective immune response against BYDV. These findings indicate that while both cultivars activate upstream MAPK signaling components in response to BYDV infection, the resistant cultivar MN10253 demonstrates stronger and more effective activation of downstream components, leading to the induction of robust defense mechanisms. First, *FRK1* is an important gene associated with plant disease resistance, and its high expression is usually considered as a plant response to infestation by pathogenic microorganisms. However, in the context of our study, even though *FRK1* is highly expressed in susceptible varieties, this does not necessarily mean that its downstream genes are not important for BYDV resistance. In fact, *FRK1* expression may interact with other regulatory mechanisms to influence the degree of activation of downstream signaling pathways. Sometimes, even if *FRK1* is highly expressed, if its downstream genes or pathways are not effectively responded to or activated, the resistance effect may still be insufficient. Therefore, the relationship between the expression level of *FRK1* and the function of its downstream genes may be more complex, and the importance of resistance cannot be judged simply by its high expression [41].

### 4.3. Differential Effects on Phytohormone Levels and Signaling Pathways

#### 4.3.1. Salicylic Acid

The SA signaling pathway plays a pivotal role in plant immunity, particularly in the defense against biotrophic pathogens [13]. Specifically, we observed typical cellular features of HR-including localized cell death, oxidative burst, and callose deposition. Resistance is associated with a rapid and effective micro-HR, which likely contributes to the observed suppression of viral movement and the absence of systemic symptoms, even in the absence of visible necrosis. This pattern aligns with what has been described in some virus-resistant genotypes, where an early and spatially restricted HR at the cellular level is sufficient to confer effective immunity without macroscopic tissue damage [14]. *NPR1*, a critical transcriptional regulator in the SA signaling pathway, interacts with the *TGA* (TGACG motif-binding factor) to activate SA-dependent plant defense responses upon pathogen attack [41]. Beyond its role in short-term immune responses, *NPR1* is also integral to establishing systemic acquired resistance, enabling plants to respond more rapidly and effectively to subsequent pathogen encounters [14]. In tea (Camellia sinensis) plants, differential gene expression analysis revealed that two *NPR1* genes, two *TGA* genes, and five PR1 genes in the SA signaling pathway were significantly upregulated, whereas only one PR1 gene was downregulated across four stages of susceptibility [42]. Similarly, in this study, we observed a significant upregulation of several DEGs encoding *NPR1* and one DEG encoding *TGA* at 48 h post-infection in the resistant oat variety MN10253, highlighting the importance of SA-dependent transcriptional regulation in mediating oat defense responses to BYDV infection.

Furthermore, the upregulation of the *PAL* gene, encoding a key enzyme in SA biosynthesis [43], at 24 and 48 h in MN10253 underscores the robust activation of the SA pathway in this variety. This likely facilitates higher SA accumulation, thereby strengthening pathogen defense. The dynamic changes in SA content provide further evidence of the enhanced defense response in MN10253. Notably, SA levels in MN10253 initially increased and peaked early, showing significantly higher concentrations at 8 h post-infection compared to the susceptible variety Qingyin 1. This early and transient accumulation of SA likely acts as a critical signal for activating downstream defense mechanisms, consistent with prior studies emphasizing the importance of timely SA accumulation in pathogen recognition and response [44,45]. Collectively, these findings underscore the central role of the SA signaling pathway in promoting oat resistance to BYDV. The enhanced activation of key components, such as *NPR1*, *TGA*, *PAL*, and their downstream targets, highlights the critical importance of timely and robust SA signaling in conferring pathogen resistance in MN10253.

#### 4.3.2. Gibberellin

The gibberellin (GA) signaling pathway is fundamental to plant growth and development but has also been increasingly recognized for its role in modulating plant defense responses against pathogens [46,47]. This dual function often results in a tradeoff between growth and defense as plants must allocate resources to prioritize one process over the other [46]. The GA signaling pathway involves three primary components: GA receptors (*GID1*), *DELLA* proteins, and downstream transcription factors. DELLA proteins serve as growth repressors, and their degradation, triggered by the formation of GA-GID1 complexes, enables plant growth [48]. Interestingly, stabilized *DELLA* proteins—due to low GA levels or impaired *GID1* activity—can enhance jasmonic acid signaling and promote plant defense against pathogens [49].

In rice (*Oryza sativa*), gibberellins have been shown to increase susceptibility to pathogens such as Xanthomonas oryzae pv. oryzae (Xoo) and Magnaporthe oryzae (Mo) [50]. Knockdown lines of genes controlling the biosynthesis of bioactive GAs, such as GA20ox and GA3ox, exhibit enhanced resistance to rice pathogens Mo and Xoo, accompanied by increased expression of defense-related genes [51]. In this study, we observed significant downregulation of the *GA3ox* gene at 8 or 24 h post-infection, accompanied by a notable decrease in GA3 levels at 48 h following BYDV infection in both oat genotypes. These findings suggest that suppressing GA biosynthesis is a common response to BYDV infection in oats. Furthermore, several DEGs encoding *GID1* and *DELLA* proteins were remarkably upregulated at 48 h in the resistant genotype MN10253, with expression levels significantly higher than those observed in the susceptible genotype Qingyin 1. The upregulation of *GID1* and *DELLA* in MN10253 highlights the potential role of stabilized DELLA proteins in mediating enhanced defense responses. Previous studies have demonstrated that stabilized *DELLA* proteins could antagonize GA-induced growth processes and, simultaneously, potentiate defense pathways, including JA signaling [49]. The enhanced expression of *GID1* and *DELLA* in MN10253 suggests that this genotype may strategically suppress GA biosynthesis to stabilize DELLA proteins, thereby reallocating resources toward defense responses against BYDV.

#### 4.3.3. Auxin

Auxin, a pivotal phytohormone regulating plant growth and development, also plays a multifaceted role in modulating plant responses to pathogen infections [52]. Generally, auxin promotes susceptibility to biotrophic pathogens by suppressing SA-mediated defense responses [53,54]. For example, *YUCCA6*, a key gene in auxin biosynthesis, has been shown to enhance plant susceptibility to pathogens [55]. Overexpression of *YUCCA6* in Arabidopsis thaliana leads to elevated auxin levels and increased susceptibility to Pseudomonas syringae, primarily due to suppression of SA biosynthesis and signaling [53]. In this study, we observed that a *YUCCA* gene was significantly downregulated at 8 h post-infection in the resistant oat genotype MN10253, accompanied by a decrease in IAA levels at 48 h. In contrast, the susceptible genotype Qingyin 1 exhibited a significant increase in IAA levels at 48 h following BYDV infection. These findings suggest that reduced auxin biosynthesis and accumulation may contribute to the resistance of MN10253, while elevated auxin levels may enhance the susceptibility of Qingyin 1 to BYDV.

Auxin signaling also appears to play a role in the differential responses between the two genotypes. In MN10253, several DEGs encoding *TIR1* (TRANSPORT INHIBITOR RESPONSE 1) and *ARF* (AUXIN RESPONSE FACTOR) were significantly downregulated following BYDV infection. This aligns with previous studies indicating that SA-mediated plant immunity can repress the expression of genes encoding auxin receptors, such as *TIR1* [56]. In *A. thaliana*, mutants with impaired auxin signaling components, such as tir1, exhibit enhanced resistance to *Pseudomonas syringae* due to elevated SA signaling [57]. Overall, the downregulation of *YUCCA*, *TIR1*, and *ARF* genes in MN10253 may reduce auxin biosynthesis and signaling, facilitating a stronger SA-mediated defense response against BYDV infection. Oats are known to produce avenacins, which confer resistance to fungal pathogens [56]; whether these metabolites also inhibit BYDV or aphid vectors remains unexplored. Future studies should quantify chorismate flux in infected oats to test whether redirection to defense metabolites complements SA-mediated immunity. This could reveal novel breeding targets to engineer ‘metabolic resistance’ against viruses.

#### 4.3.4. Abscisic Acid

An increase in endogenous ABA levels has been observed in plants infected with various pathogens [58,59]. However, ABA plays multifaceted roles in plant–pathogen interactions, with its effects generally depending on the stage of infection [60,61]. At early stages of infection, ABA can positively regulate plant defense by mediating stomatal closure and callose deposition, thereby limiting pathogen entry. In contrast, at later stages, ABA can negatively affect plant defense by suppressing ROS production and antagonizing the SA and JA signaling pathways [62,63]. In this study, a slight increase in ABA levels was observed at 8 h post-infection in the resistant genotype MN10253. In contrast, a pronounced increase in ABA levels occurred at 48 h in the susceptible genotype Qingyin 1. This elevated ABA content in Qingyin 1 may be associated with the upregulation of genes involved in ABA biosynthesis, such as *NCED* and *AAO3*. Previous studies have reported that overexpression of *NCED* genes in *A. thaliana* enhanced susceptibility to *Pseudomonas syringae* infection, further supporting the link between elevated ABA biosynthesis and pathogen susceptibility [64].

Additionally, the ABA signaling pathway appears to contribute to the susceptibility of Qingyin 1 to BYDV. Recent research has shown that the ABA central regulatory complex PP2C-SnRK2 modulates ABA-dependent defense responses against viral pathogens [65]. For instance, in *Nicotiana benthamiana*, *PP2C4* interacts with and constitutively inhibits *SnRK2.3/2.4*, suppressing the expression of ABA-responsive genes such as NbABF2, NbABF4, and NbRAB18. Notably, knockout of *NbPP2C4* reduced infection by *Tomato spotted wilt orthotospovirus* [65]. In this study, we observed significant upregulation of three PP2C genes and concurrent downregulation of two *SnRK2* genes and three *ABF* genes at 2 h post-infection in Qingyin 1. These findings suggest that the elevated ABA content and altered ABA signaling in Qingyin 1 may contribute to its susceptibility to BYDV infection.

### 4.4. Limitations and Future Perspectives

Although this study provides comprehensive insights into the transcriptomic and phytohormone-mediated defense responses to BYDV infection in oats, several methodological limitations should be noted. HR typically occurs in localized areas at the sites where aphids transmit BYDV. As total RNA was extracted from entire leaves, the transcriptomic signal from these minute HR foci may have been diluted, potentially underrepresenting highly localized defense reactions. Nevertheless, our aim was to characterize the overall defense dynamics at the tissue level, encompassing both local and systemic responses. Given that systemic signals such as salicylic acid can trigger transcriptional reprogramming throughout the leaf, bulk RNA sequencing remains suitable for capturing the integrated defense responses of resistant and susceptible cultivars. Future work combining fine-scale sampling at infection sites with single-cell or spatial transcriptomics could more precisely resolve localized HR responses and virus–host interaction zones, thereby complementing the systemic view presented in this study.

## 5. Conclusions

In summary, this study reveals a more robust immune response, characterized by strong activation of PTI and ETI pathways and higher expression of defense-related genes, particularly those associated with MAPK signaling and SA pathways in resistant cultivar MN10253. The early accumulation of SA in MN10253 likely triggered enhanced immune responses, while downregulation of auxin and ABA-related genes helped reduce hormonal antagonism, further boosting resistance. In contrast, the susceptible cultivar Qingyin 1 exhibited delayed or weaker immune activation and elevated levels of auxin and ABA, which may interfere with SA-mediated immunity, leading to increased susceptibility. These findings emphasize the critical role of precise hormonal regulation and the coordination of defense pathways in disease resistance. Overall, this research provides valuable insights into the BYDV–oat interaction and lays the groundwork for future oat breeding programs aimed at improving disease resistance. Further exploration of gene-editing techniques and hormonal pathway manipulation could significantly enhance resistance in oats.

## Data Availability

The raw RNA sequencing data were submitted to the NCBI database with the BioProject ID: PRJNA1186746. All data will be made available upon request.

## Supplementary Materials

The following supporting information can be downloaded at: https://www.mdpi.com/article/10.3390/plants14203229/s1, Table S1: Sequencing data statistics.
