# Supplementary material for: The Phytohormone Signaling Pathway and Immunity Responses to BYDV Infection in Resistant and Susceptible Oat Cultivars
Source: Plants (Basel). 2025 Oct 21;14(20):3229. doi: 10.3390/plants14203229 (PMC12566944; doi:10.3390/plants14203229)
Supplement: Supplementary file 1 [file plants-14-03229-s001.zip › plants-3887853-supplementary.pdf]

Table S1 Sequencing data statistics

#SampleID	ReadSum	BaseSum	GC(%)	N(%)	Q20(%)	Q30(%)
M0-1	32198714	9611425158	56.41	0	98.11	94.74
M0-2	25142550	7516658268	55.72	0	98.12	94.7
M0-3	23617223	7058337298	55.85	0	98.04	94.51
M2-1	30333606	9056253306	55.06	0	98.16	94.81
M2-2	24256935	7256001070	56.4	0	97.96	94.33
M2-3	30550977	9136265572	54.83	0	97.95	94.26
M24-1	26342262	7873382806	55.96	0	98.01	94.45
M24-2	28790298	8607158188	55.13	0	97.89	94.15
M24-3	31929055	9536922782	56.19	0	98.04	94.54
M48-1	24497424	7325676526	54.02	0	97.89	94.13
M48-2	27717969	8276561934	54.53	0	98.01	94.48
M48-3	30300496	9061348920	54.74	0	98.02	94.46
M8-1	20716188	6194182610	55.84	0	97.98	94.36
M8-2	28149140	8418374702	55.58	0	97.97	94.39
M8-3	28504686	8515839792	56.1	0	97.97	94.35
Q0-1	22877678	6845780154	55.96	0	97.97	94.35
Q0-2	28175397	8423284088	56.55	0	98.17	94.88
Q0-3	26217763	7829505004	55.12	0	97.97	94.35
Q2-1	31471260	9406693498	54.96	0	97.91	94.21
Q2-2	27850992	8326824162	55.28	0	97.88	94.1
Q2-3	29362953	8778406498	55.93	0	97.8	94.12
Q24-1	26270155	7850495662	55.14	0	97.73	93.91
Q24-2	25061440	7498699856	55.43	0	97.45	93.24
Q24-3	21271041	6361967956	54.89	0	97.46	93.27
Q48-1	22540244	6745150182	54.27	0	97.33	92.99
Q48-2	20969588	6274214146	53.91	0	97.68	93.76
Q48-3	19546809	5851493286	54.75	0	97.65	93.64
Q8-1	20009391	5989698866	55.07	0	97.54	93.45
Q8-2	22246424	6651107652	54.39	0	97.81	94.09
Q8-3	22396871	6702169162	55.5	0	97.69	93.83
